# Multi-height metasurface for wavefront manipulation fabricated by direct laser writing lithography

**DOI:** 10.1515/nanoph-2023-0268

**Published:** 2023-07-31

**Authors:** Fan Ye, Mike Pivnenko, Huiyu Huang, Xin Chang, Lee Robinson, Youdou Zheng, Yi Shi, Daping Chu

**Affiliations:** Center for Photonic Devices and Sensors, University of Cambridge, Cambridge CB3 0FA, UK; School of Electronic Science and Engineering Nanjing University, Nanjing, Jiangsu 210093, P.R. China

**Keywords:** dielectric metasurfaces, direct laser writing, phase modulation

## Abstract

We introduce two types of dielectric metasurfaces, consisting of 3 × 3 regions, which manipulate wavefront by different feature heights. Both polarization-dependent and polarization-independent metasurfaces are realized for phase depth of 0 ∼ 2π at 1550 nm, with considerable average transmittance of 80.1 and 85.1 %, respectively. The phase modulation capability can be extended over a broadband range of 1460.1–1618.0 nm for optical communications, by carefully designing nanofeature sizes. Moreover, the entire metasurfaces with nanofeatures of varying heights can be fabricated in a single process by using direct laser writing with high-precision, which is beneficial for mass production and promising in developing efficient and ultracompact devices.

## 1 Introduction

Metasurface is a planar structure consisting of arrays of subwavelegth optical elements (metallic or dielectric) which can alter phase [1–4], amplitude [5, 6] and polarization [7, 8] of incident light. The primary function of the ultra-thin patterned meta device is to modulate the phase thereby shaping the wavefront of incoming light based on the spatially different distribution of the scatterers [9, 10]. By optimizing geometrical parameters (shape, size, orientation, and pitch) of periodic unit cells, arbitrary phase shifts can be achieved as along with the transmittance [9, 11, 12]. Such manipulation is not limited to a specific wavelength but can be extended to a broadband range through proper dispersion designs of various regions. The favorable characteristic enables versatile functionalities in holograms [13–15], flat lenses [2, 13, 16], quarter and half waveplates [6, 17], anomalous refraction [18] and orbital angular momentum (OAM) [19].

Plasmonic metasurfaces often suffer from low efficiency (25 %) due to weak coupling between cross-polarized fields and the incident light [20, 21]. By contrast, dielectric metasurfaces, free from Ohmic losses in metal, have emerged as a promising candidate for high efficiency wavefront manipulation and have attracted enormous attention in these years. Asymmetric building blocks are typically applied to achieve 0 to 2π phase control due to the design flexibility [9, 22], while the symmetric are commonly used for reflectionless by simultaneous excitation of electric and magnetic dipole resonances [23, 24]. However, conventional methods like E-beam lithography (EBL) and focused ion beam (FIB) to fabricate all-dielectric metasurfaces is cost prohibitive and time-consuming over large scale, and can only fabricate nanoparticles of the same height in a single process [25, 26]. Additionally, the process is normally accompanied with deposition, lithography and etching, which will bring about side erosion and finally lead to a less steep and less straight sidewall. To overcome these limitations, various types of 3D printing technologies are widely utilized to fabricate dielectric metasurfaces for their convenience and flexibility [26, 27]. Among these sequential 3D printing methods, direct laser writing (DLW) stands out as an effective additive technique capable of creating high-precision and diverse shaped lattice structures [28–31].

This study presents two types of multi-height metasurfaces (polarization-dependent and polarized–independent) in 3 × 3 regions, numerically and experimentally illustrating their phase modulation ability around 1550 nm. The proposed metasurfaces were fabricated through DLW based on two-photon polymerization (2 PP) using Nanoscribe Photonic Professional GT instrument. The rectangular nanopillars and cylindrical nanodisks of 9 different sizes were simultaneously grown on a silica fused substrate in corresponding areas, leveraging the various height processing capabilities of 3D printing. The entire metasurface achieves a broadband 0 to 2π phase shift from 1460.1–1618.0 nm across the telecommunication regime. Furthermore, the average transmittances of the two metasurfaces are 80.1 % and 85.1 %, respectively. These meta devices offer flexible phase tuning ability and high efficiency, making them suitable for implementing vectorial holograms and arbitrary OAM functionalities.

## 2 Analysis and simulation

### 2.1 Polarization-dependent metasurface

Dielectric metasurfaces composed of silicon nanoparticles provide ranges of functionalities due to the low absorption loss. Nonetheless, the application of those based on single localized mode is restricted in reflection geometry due to the sharp increase in reflectance at the resonance wavelength [32]. Therefore, optimizing the size (height, width, length, and pitch) of individual nanopillar is crucial to control the excitation of electric and magnetic dipolar resonances simultaneously, realizing reflectionless behavior at the targeted wavelength. To calculate the accumulated phase shift *φ* (neglecting Fabry–Perot effects) as light passes through the nanopillar, the following equations are used [33]:

$$\Phi =2\pi \cdot n_{\text{eff}}\cdot H/\lambda ,$$

$$n_{\text{eff}}=n_{r}\cdot W\cdot L/P^{2}+n_{\text{air}}\left(1-W\cdot L/P^{2}\right),$$

where *n*_eff_ is the effective refractive index, *H*, *W*, *L* denote the height, width, and length of the nanopillar, and *P* is the pitch between adjacent nanopillars. By these formulae, we can see that phase shift *φ* can be modified by adjusting *H*, *W* and *L*. In order to achieve the wavefront control through a multi-height metasurface around the telecommunication, geometry parameters optimization was performed using commercially available software CST MICROWAVE STUDIO.

**Figure 1:**
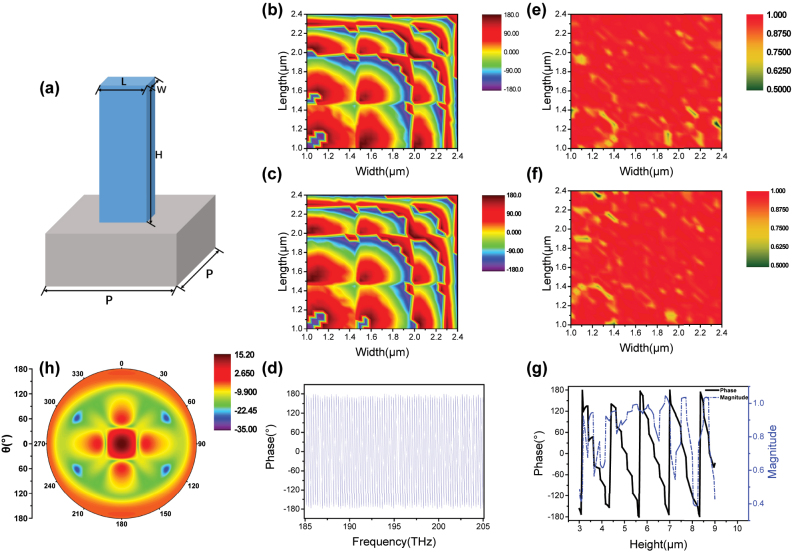
Schematic and simulation results of structure. (a) Schematic of a rectangular nanopillar. Phase shift with varying width and length under (b) *x*-polarized, (c) *y*-polarized, (d) in range of 185 ∼ 205 THz. Transmission amplitude with width and length under (e) *x*-polarized, (f) *y*-polarized. (g) Phase shift and transmission amplitude with varying height. (h) Farfield when monitor is 500 μm away from the metasurface. All results are under incidence at 1550 nm.

As schematically demonstrated in Figure 1(a), structure of single unit with rectangular nanopillars, composed of resin (*n*_*r*_ = 1.53), standing on 700-μm silica fused substrate (*n*_*s*_ = 1.444). As illustrated in Figure 1(b) and (c), when *H* and *P* are fixed in 5.56 and 2.5 μm, the phase shift *φ*_*x*_ and *φ*_*y*_ under the *x*- and *y*-polarized incidence at 1550 nm are recorded respectively as we gradually varied the *W* and *L*. The phase shifts *φ*_*x*_ and *φ*_*y*_ cover 0 to 2π as *W* and *L* ranges from 1.0 to 2.4 μm. The phase shift of the incidence at different frequency from 185 to 205 THz is shown in Figure 1(d). The result reveals a large amount of Mie-type scattering resonances resulting from both induced electric and magnetic dipoles as designed within the range [11, 34], which explains the dramatic changes in phase shifts *φ* with slight variation in *W* and *L* under both polarizations. On the other hand, the tuning ranges still approach 2π at 185 and 205 THz, details can be looked up in Supplementary Material, suggesting the free phase modulation can be achieve in broadband from 185 to 205 THz, corresponding to 1460.1–1618.0 nm.

The transmission amplitude, represented by the square root of the intensity transmittance, is defined by $\left|t\right|=\left|t_{s}\right|/\sqrt{n_{s}}$ [9], where *t*_*s*_ denotes the simulated transmission coefficient of the interface from the silica fused substrate across the resin to the air. The transmission amplitudes *t*_*x*_ and *t*_*y*_ under *x*- and *y*-polarized are plotted in Figure 1(e) and (f). It can be observed that both the phase shifts and transmission amplitudes are polarization-dependent but have symmetry about the line *W* = *L* due to the anisotropic rectangular-shaped elements. It is worth mentioning that the transmission amplitude is basically above 77 % across the entire range, indicating that full phase modulation can be achieved while ensuring considerably high transmission. Nevertheless, modulating the phase by varying *W* and *L* has a fundamental flaw – the phase shift *φ* can change drastically with small fabrication errors. By contrast, as shown in Figure 1(g), when *W* and *L* is set to be 1.4 and 2.0 μm, measured under *x*-polarized at 1550 nm, the phase shift decreases linearly with the height within one cycle (such as 5.68–6.96 μm) of change while the transmission can drop to 39 % in some specific height. Hereby, it is more reliable to modulate phase through the optimized height and then improve further by altering *W* and *L*. In addition, although we know the propagation phase is wavelength-dependent, such height tuning of phase shift is also effective for a fixed wavelength. According to simulation results, we specified the respective sizes of 9 areas, and the parameters are listed in Table 1. *H* of nanopillars varies between 4.36 and 7.76 μm, *W* and *L* are from 1.0 to 2.4 μm in Regions I ∼ IX. In this way, the *φ*_*x*_ in adjacent regions are set at an approximately 45° interval and maintain *t*_*x*_ at a relatively high level even at resonance. For the *φ*_*y*_ and *t*_*y*_ under *y*-polarized, they are all different from but relatively close to those which are under *x*-polarized except for the transmission amplitudes *t*_*y*_ of Region IV (∼46.20 %). Figure 1(h) depicts the simulated far-field of Region IX when placing the monitor 500 μm away from the metasurface. Most of intensity gathers in the center, which is the component transmitting directly through the metasurface. There is also a slight distribution at a deflection angle of *θ* = 59°, attributed to the first-order diffraction.

**Table 1:** Parameters of nanopillars from 9 regions in polarization-dependent metasurface.

Region	*H* (μm)	*W* (μm)	L (μm)	$\varphi_{x}\left({}^{\circ}\right)$	$t_{x}\left(\text{\%}\right)$	$\varphi_{y}\left({}^{\circ}\right)$	$t_{y}\left(\text{\%}\right)$
I	4.36	1.5	1.4	−174.73	79.4	176.29	72.19
II	5.56	1.3	2.2	−134.64	98.6	−141.15	92.80
III	6.56	1.5	1.2	−93.06	92.5	−88.33	98.24
IV	7.76	1.4	2.0	−44.39	88.1	−61.53	46.20
V	4.88	1.0	1.6	2.78	80.6	10.00	85.50
VI	7.48	1.4	2.4	48.24	80.2	52.36	100
VII	6.04	1.4	1.8	89.02	94.8	84.71	95.59
VIII	7.04	1.4	2.0	140.13	100	149.42	86.87
IX	5.60	1.4	1.8	178.62	93.8	176.72	99.69

### 2.2 Polarization-independent metasurface

**Figure 2:**
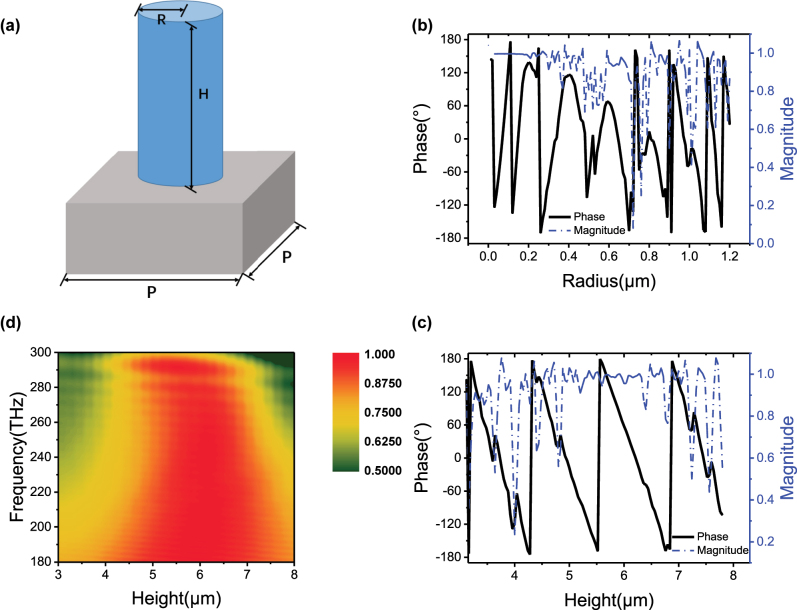
Schematic and simulation results of structure. (a) Schematic of a cylindrical nanopillar. Phase shift and transmission amplitude with varying (b) radius and (c) height, under an incidence light at 1550 nm. (d) Transmission amplitude with height in range of 180–300 THz.

Various functionalities, such as resonant forward scattering, broad-band reflector and spontaneous emission control, have also been demonstrated with isotropic cylindrical-shaped metasurfaces. Similarly, Periodic structures composed of nanodisks, also known as Huygens’ metasurfaces [34–36], have been wildly utilized due to their extremely high transmittance. In line with these purposes, the metasurface consisting of different-sized nanodisks in 3 × 3 regions has also been thoroughly studied, and the effective refractive index can be expressed as:

$$n_{\text{eff}}=n_{r}\cdot \pi \cdot R^{2}/P^{2}+n_{\text{air}}\left(1-\pi \cdot R^{2}/P^{2}\right),$$

where *R* denotes the radius of nanodisks. Figure 2(a) demonstrates the structure of single unit with cylindrical nanodisk, made of resin polymer, standing on silica fused substrate. As shown in Figure 2(b), when *H* and *P* are fixed at 5.60 and 2.5 μm, respectively, the phase shift *φ* and transmission amplitude *t* are both recorded. By altering radius *R*, we achieve 0 to 2π phase shift when radius is above 0.7 μm, however, the transmission amplitude is low at some points within this range. Moreover, the phase shift changes linearly only in small ranges, such as 1.08–1.16 μm, making it difficult to fabricate the optimized radii with precision. Figure 2(c) demonstrates that, when the radius is 1.1 μm, the phase shift decreases linearly between 4.32–5.52 and 5.56–6.84 μm, and the transmission amplitude is mostly above 80 % except for a few points. Transmission amplitude with respect to height for a wider range of incident light frequencies (180 ∼ 300 THz) is illustrated in Figure 2(d). The transmission amplitude is above 70 % between 180–280 THz when height in the range of 4.20–7.20 μm, indicating its broad-band availability. At the same time, results of the phase shift and transmission amplitude are independent of the polarization of the incidence. Since the transmittance is high enough, we only varied the heights of nanodisks between 4.28–6.52 μm with the same radius of 1.1 μm in 9 areas, and the parameters are listed in Table 2. At arbitrary polarization, the phase shift *φ* interval is roughly 45° between the adjacent. The average transmission amplitude *t* is higher than that of the polarization-dependent metasurface. Especially for Regions B, C, F, G, and H, the amplitudes approach 100 %, which proves reflectionless thanks to electromagnetically dual-symmetric behavior.

**Table 2:** Parameters of nanodisks from 9 regions in polarization-independent metasurface.

Region	A	B	C	D	E	F	G	H	I
*H* (μm)	4.28	5.40	6.52	5.08	6.20	4.72	4.60	5.72	5.56
$\varphi \left({}^{\circ}\right)$	−174.91	−135.51	−87.99	−43.78	3.28	45.4	92.56	136.98	179.42
$t\left(\text{\%}\right)$	85.8	99.5	99.8	96.2	98.9	100	100	99.6	97.4

### 2.3 Fabrication

**Figure 3:**
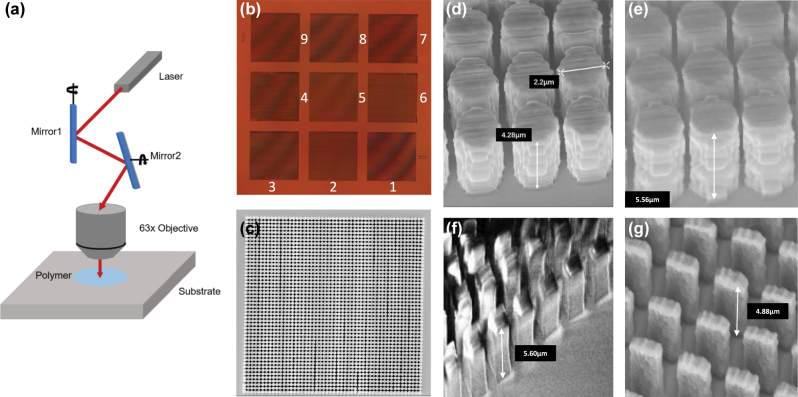
Details of fabricated metasurfaces. (a) Schematic of 2 PP fabrication technology. (b) Microscope photo of the polarization-dependent metasurface. (c) SEM image of Region I in polarization-independent metasureface (top view). (d), (e), (f), (g) SEM images of Regions A, I, IX, V in polarization-independent and -dependent metasurefaces, respectively (side view).

Two gradient height metasurfaces were achieved by using the commercial prototyping instrument (Nanoscribe Photonic Professional GT) based on 2 PP in a tightly focused femtosecond laser beam [37, 38], as shown in Figure 3(a). The nanopillars with 9 different sizes were fabricated within separated 3 × 3 areas throughout the entire metasurfaces. The polymer based metasurfaces were built on a fused silica substrate with IP-Dip2 resist on. For exposure, high numerical aperture (NA1.4) objective (Zeiss Plan-Apochromat 63×) was applied in immersion configuration. The laser beam was working on Galvo scanning mode, and to fit various sizes of pillars, optimized scan speed 10,000 μm s^−1^, laser power of 32.5 and 45 mW for rectangular-shaped and cylindrical-shaped were chosen, respectively. Small slicing and hatching (laser movement step in longitudinal and transverse direction were set to be 0.04 and 0.1 μm to enable high aspect ratios and maintain mechanical strength of the polymer nanopillars simultaneously. Afterwards, the exposed samples were developed in propylene glycol monomethyl ether acetate (PGMEA) for 15 min and then immersed in isopropanol for 5 min. Eventually, the fabricated polymer sample would be dried in nitrogen for 2 min. The two fabricated samples both contain 9 square areas with same size of 125 × 125 μm, 25 μm spacing between adjacent square regions, and the size of entire metasurfaces are 425 × 425 μm, as exhibited in Figure 3(b), which is photo of polarization-dependent metasurface taken by the optical microscope. Figure 3(c) is the SEM top view of Region I after deposited a layer of Au to enable conductivity for the photo purpose. The resolution along the height direction is the same as the small slicing of 0.04 μm. The SEM side views of Regions A, I, IX, and V are, respectively, depicted in Figure 3(d)–(g), which indicates the shape and height variation in two metasurfaces, and the precision of fabrication through 2 PP as well.

## 3 Results

**Figure 4:**
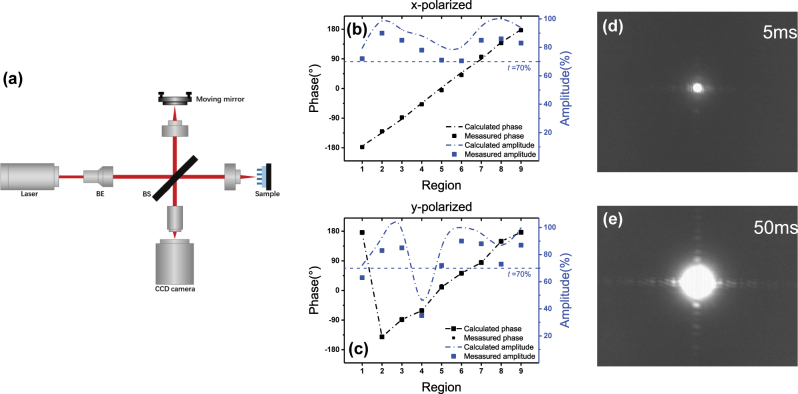
Results of polarization-dependent metasurface. (a) The optical setup for phase measurement. Comparison between simulated and experimental results of polarization-dependent metasurface (b) under *x*-polarized, (c) under *y*-polarized at 1550 nm. The far-field of the metasurface at an exposure time of (d) 5 ms, (e) 50 ms.

To characterize the performance of the designed metasurface, we measured the phase shift of different regions using Michelson interferometer at 1550 nm as shown in Figure 4(a). The experimental setup consists of light source, beam enhancement optics, beam splitter, detector, and the sample mounted on a fixed mirror. Index matching liquid was applied to arrange the reflection mode so that its phase shift can be obtained by analyzing the interference fringes. During the experiment, we varied the angle between telecommunication transmitter and receiver from 0° to 90° to achieve horizontal and vertical polarization, respectively.

The simulated and experimental results of polarization-dependent metasurface are recorded in Figure 4(b) and (c). The measured phase (black squares) matched well with the calculated ones (black dash-dot lines), and measured amplitude (blue squares) are slightly smaller than calculated ones (blue dash-dot lines). For *x*-polarized incidence at 1550 nm, the phase shift *φ* in Region I is −178.2° and rises in generally equal intervals to 177.5° in Region IX, which indicates the metasurface achieved 2π phase shift and can be used to realize arbitrary wavefront manipulation. Moreover, the transmittance across the whole plate is all over 70.5 %, justifying a considerably high efficiency of the device; For *y*-polarized incidence, phase shift of Region I deviates to 173.4° because it is near the resonance, two regions have transmittance fall below 70 %, and the amplitude of Region IV drops *drastically to 35 %, which can be employed in* polarization selection. Figure 4(d) and (e) depict the far-field of Region IX when the sensor is 100 mm away from the sample at exposure times of 5 ms and 50 ms, respectively. Only zero order light can be seen at short exposure time, and the higher orders of diffraction appear as exposure increases, which is in accordance with simulation result in Figure 1(h).

**Figure 5:**
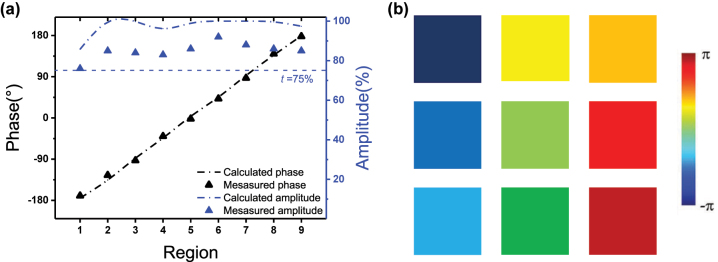
Results of polarization-independent metasurface. (a) Comparison between simulated and experimental results of polarization-independent metasurface under arbitrary polarization. (b) Illustration of rainbow like pattern of phase shift in metasurface.

In terms of polarization-independent metasurface, the results are shown in Figure 5(a). The experimental phase shifts (black triangles) is also in good agreement with simulated ones (black dash-dot lines), and the transmittance performance, with all values above 76 % and an average of 85.1 %, is better than those in rectangular-shaped metasurface owing to perfect matching of the electric and magnetic polarizabilities of isotropic nanodisks. According to linear modulation of phase in adjacent regions, the phase shifts in entire metasurface can achieve such palette-like pattern as drawn in Figure 5(b). Furthermore, it is very easy to achieve mass production for this metasurface through DLW [39, 40], enabling promising prospects in low-loss and compact devices in telecom regime.

## 4 Conclusions

In summary, two metasurfaces fabricated through DLW were presented, each consisting of 3 × 3 regions with rectangular and cylindrical nanopillars of different sizes, respectively. By varying the height of nanopillars linearly, both metasurfaces can achieve a full range of 2π phase shift at 1550 nm. At the same time, an average transmission of 80.1 % is obtained for the polarization-dependent case by adjusting the width and length of the rectangular nanopillars in each region. Transmissions for polarization-independent metasurface with cylindrical nanopillars can be as high as 85.1 % on average with the same radius of 1.1 μm, by controlling of the electric and magnetic dipole resonances in the symmetric disc-like features. Finally, such 3D printed metasurfaces can benefit from getting away from complex and time-consuming fabrication processes and bring in promising perspectives for important applications such as OAM, hologram and low-loss devices.

## Supplementary Material

Supplementary Material Details

## References

[1] Yu N., Genevet P., Kats M. A. (2011). Light propagation with phase discontinuities: generalized laws of reflection and refraction. *Science*.

[2] Aieta F., Genevet P., Kats M. A. (2012). Aberration-free ultrathin flat lenses and axicons at telecom wavelengths based on plasmonic metasurfaces. *Nano Lett*..

[3] Huang L., Cheng X., Muhlenbernd H. (2012). Dispersionless phase discontinuities for controlling light propagation. *Nano Lett*..

[4] Zhang X., Tian Z., Yue W. (2013). Broadband terahertz wave deflection based on C‐shape complex metamaterials with phase discontinuities. *Adv. Mater.*.

[5] Papakostas A., Potts A., Bagnall D. M., Prosvirnin S. L., Coles H. J., Zheludev N. I. (2003). Optical manifestations of planar chirality. *Phys. Rev. Lett.*.

[6] Yu N., Aieta F., Genevet P., Kats M. A., Gaburro Z., Capasso F. (2012). A broadband, background-free quarter-wave plate based on plasmonic metasurfaces. *Nano Lett*..

[7] Liu L., Zhang X., Kenney M. (2014). Broadband metasurfaces with simultaneous control of phase and amplitude. *Adv. Mater.*.

[8] Sun S., He Q., Xiao S., Xu Q., Li X., Zhou L. (2012). Gradient-index meta-surfaces as a bridge linking propagating waves and surface waves. *Nat. Mater.*.

[9] Zhang H., Zhang X., Xu Q. (2018). High‐efficiency dielectric metasurfaces for polarization‐dependent terahertz wavefront manipulation. *Adv. Opt. Mater.*.

[10] Xu Y., Li Q., Zhang X. (2019). Spin-decoupled multifunctional metasurface for asymmetric polarization generation. *ACS Photonics*.

[11] Chong K., Staude I., Staude A. (2015). Polarization-independent silicon metadevices for efficient optical wavefront control. *Nano Lett.*.

[12] Zhang H., Zhang X., Xu Q. (2018). Polarization-independent all-silicon dielectric metasurfaces in the terahertz regime. *Photonics Res.*.

[13] Hu D., Wang X., Feng S. (2013). Ultrathin terahertz planar elements. *Adv. Opt. Mater.*.

[14] Wang B., Quan B., He J. (2016). Wavelength de-multiplexing metasurface hologram. *Sci. Rep.*.

[15] Wang Q., Zhang X., Xu Y. (2016). Broadband metasurface holograms: toward complete phase and amplitude engineering. *Sci. Rep.*.

[16] Wang Q., Zhang X., Xu Y. (2015). A broadband metasurface-based terahertz flat-lens array. *Adv. Opt. Mater.*.

[17] Ding F., Wang Z., He S., Shalaev V. M., Kildishev A. V. (2015). Broadband high-efficiency half-wave plate: a supercell-based plasmonic metasurface approach. *ACS Nano*.

[18] Zhu H., Semperlotti F. (2016). Anomalous refraction of acoustic guided waves in solids with geometrically tapered metasurfaces. *Phys. Rev. Lett.*.

[19] Zhang H., Kang M., Zhang X. (2017). Coherent control of optical spin‐to‐orbital angular momentum conversion in metasurface. *Adv. Mater.*.

[20] Monticone F., Estakhri N. M., Alù A. (2013). Full control of nanoscale optical transmission with a composite metascreen. *Phys. Rev. Lett.*.

[21] Arbabi A., Faraon A. (2017). Fundamental limits of ultrathin metasurfaces. *Sci. Rep.*.

[22] Luo X., Hu Y., Ou X. (2022). Metasurface-enabled on-chip multiplexed diffractive neural networks in the visible. *Light: Sci. Appl.*.

[23] Fu Y. H., Kuznetsov A. I., Miroshnichenko A. E., Yu Y. F., Luk’yanchuk B. (2013). Directional visible light scattering by silicon nanoparticles. *Nat. Commun.*.

[24] Person S., Jain M., Lapin Z., Sáenz J. J., Wicks G., Novotny L. (2013). Demonstration of zero optical backscattering from single nanoparticles. *Nano Lett*..

[25] Li N., Xu Z., Dong Y. (2020). Large-area metasurface on CMOS-compatible fabrication platform: driving flat optics from lab to fab. *Nanophoton*.

[26] Su V. C., Chu C. H., Sun G., Tsai D. P. (2018). Advances in optical metasurfaces: fabrication and applications. *Opt. Express*.

[27] Li H., Wang G. M., Hu G., Cai T., Qiu C. W., Xu H. X. (2020). 3D‐printed curved metasurface with multifunctional wavefronts. *Adv. Opt. Mater.*.

[28] Ren H., Fang X., Jang J., Bürger J., Rho J., Maier S. A. (2020). Complex-amplitude metasurface-based orbital angular momentum holography in momentum space. *Nat. Nanotechnol.*.

[29] Chen C., Liu Y., Jiang Z. Y. (2022). Large-area long-wave infrared broadband all-dielectric metasurface absorber based on markless laser direct writing lithography. *Opt. Express*.

[30] Fanyaeu I., Mizeikis V. (2016). Fabrication of metasurface-based infrared absorber structures using direct laser write lithography. *Proc. SPIE*.

[31] Conrads L., Honné N., Ulm A. (2023). Reconfigurable and polarization‐dependent grating absorber for large‐area emissivity control based on the plasmonic phase‐change material In_3_SbTe_2_. *Adv. Opt. Mater.*.

[32] Fedotov V. A., Rose M., Prosvirnin S. L., Papasimakis N., Zheludev N. I. (2007). Sharp trapped-mode resonances in planar metamaterials with a broken structural symmetry. *Phys. Rev. Lett.*.

[33] Khorasaninejad M., Capasso F. (2017). Metalenses: versatile multifunctional photonic components. *Science*.

[34] Manuel D., Staude I., Falkner M. (2015). High‐efficiency dielectric Huygens’ surfaces. *Adv. Opt. Mater.*.

[35] Staude I., Schilling J. (2017). Metamaterial-inspired silicon nanophotonics. *Nat. Photonics*.

[36] Leitis A., Heßler A., Wahl S. (2020). Huygens’ metasurfaces: all‐dielectric programmable Huygens’ metasurfaces. *Adv. Funct. Mater.*.

[37] Dottermusch S., Busko D., Langenhorst M., Paetzold U. W., Richards B. S. (2019). Exposure-dependent refractive index of Nanoscribe IP-Dip photoresist layers. *Opt. Lett.*.

[38] Žukauskas A., Matulaitienė I., Paipulas D., Niaura G., Malinauskas M., Gadonas R. (2015). Tuning the refractive index in 3D direct laser writing lithography: towards GRIN microoptics. *Laser Photonics Rev.*.

[39] Yoon G., Tanaka T., Zentgraf T., Rho J. (2021). Recent progress on metasurfaces: applications and fabrication. *J. Phys. D*.

[40] Ruiz de Galarreta C., Casquero N., Humphreys E. (2022). Single-step fabrication of high-performance extraordinary transmission plasmonic metasurfaces employing ultrafast lasers. *ACS Appl. Mater. Interfaces*.

