# Supplementary material for: Multi-height metasurface for wavefront manipulation fabricated by direct laser writing lithography
Source: Nanophotonics. 2023 Jul 31;12(17):3435–42. doi: 10.1515/nanoph-2023-0268 (PMC11501213; doi:10.1515/nanoph-2023-0268)
Supplement: Supplementary file 1 — Supplementary Material Details [file j_nanoph-2023-0268_suppl_001.docx]

Supplementary material

Figure S1 demonstrates the calculated and measured phase shift in 9 regions under incidence at 185 THz (1618.0 nm) and 205 THz (1460.1 nm). For 185 THz, the measured phase shifts of 9 regions in polarization-dependent metasurface are between -175~177°, and for 205 THz, phase shifts are between -173~177°, whose tuning ranges are 352° and 350° respectively, both approaching 2π. Therefore, we believe phase modulation capability can be extended over a broadband range of 1460.1~1618.0 nm for optical communications.


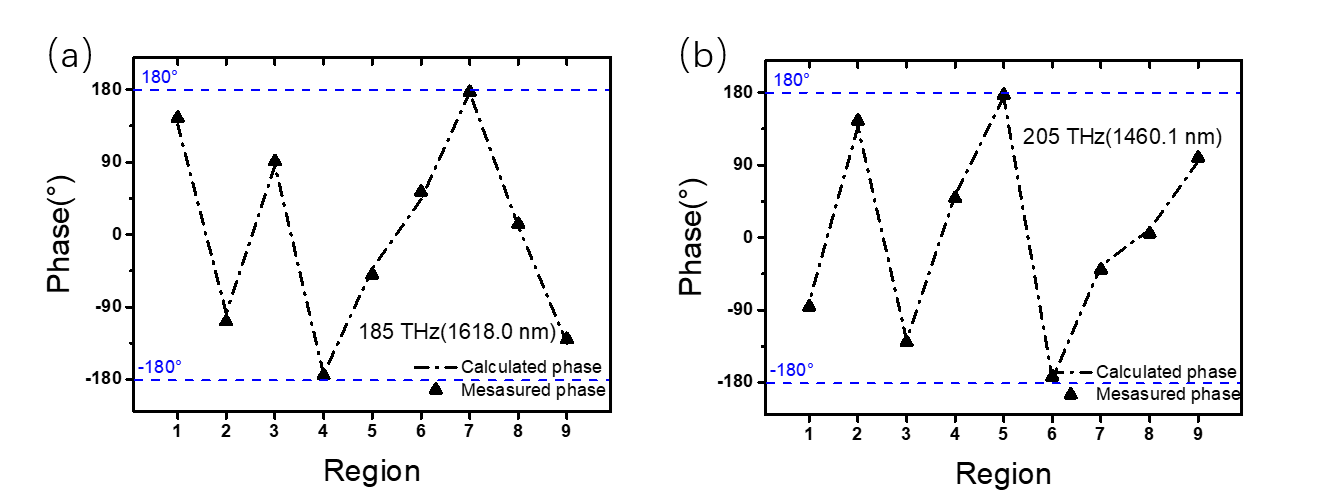


**Figure S1:** Simulated and experimental phase shifts of 9 regions in polarization-dependent metasurface under (a) 185 THz, (b) 205 THz.
